# Local vancomycin administration in Orthopaedic Surgery - A systematic review of comparative studies^☆^

**DOI:** 10.1016/j.jor.2024.03.040

**Published:** 2024-04-10

**Authors:** Darius L. Lameire, Jack Soeder, Hassaan Abdel Khalik, Ellie Pinsker, Nipun Atri, Amir Khoshbin, Lenny Radomski, Amit Atrey

**Affiliations:** a Division of Orthopaedic Surgery, University of Toronto, Toronto, Ontario, Canada; b Division of Orthopaedic Surgery, McMaster University, Hamilton, Ontario, Canada; c St. Michael's Hospital, University of Toronto, Toronto, Ontario, Canada; d Department of Internal Medicine, Division of Infectious Diseases, Rush University Medical Centre, Chicago, Illinois, USA; e Division of Orthopaedic Surgery, St. Michael's Hospital, University of Toronto, Toronto, Ontario, Canada

**Keywords:** Vancomycin, Local, Powder, Infection, Topical, Orthopaedic

## Abstract

**Background:**

There is still controversy surrounding the routine use of vancomycin locally in primary orthopaedic surgery procedures. Therefore, the aim of this review is to assess how local vancomycin impacts the rates and microbiology of surgical site infections.

**Methods:**

A systematic electronic search of MEDLINE, EMBASE, and Web of Science was carried out for all comparative studies comparing locally applied vancomycin to control for primary orthopaedic surgery procedures published before August 14, 2022.

**Results:**

A total of 61 studies with 65,671 patients were included for analysis. Forty-six studies used vancomycin powder, 12 studies with grafts soaked in vancomycin, two studies used vancomycin irrigation, and one study administered vancomycin interosseously. There were 15 studies (of 26) in spine surgery, five (of 14) in arthroplasty, ten (of 11) in sports medicine, and two (of five) in trauma surgery that found statistically significant decreases in overall infection rates when applying local vancomycin. Only one study (in spine surgery) found significant increases in infection rates with local vancomycin application. For spine surgery, local vancomycin application had the greatest proportion of gram-negative bacteria (40.7%) isolated compared to *S. aureus* (42.4%) in controls. In arthroplasty and trauma surgery, there were increases in the proportions of gram-negative bacteria when vancomycin was added. There were no reported systemic adverse reactions associated with local vancomycin use in any of the studies.

**Conclusion:**

Applying local vancomycin during primary orthopaedic surgery procedures may reduce the rates of infections in multiple different orthopaedic specialties, particularly in spine surgery and sports medicine. However, careful consideration should be applied when administering local vancomycin during specific orthopaedic procedures given the heterogeneity of included studies and breadth of surgeries included in this review.

**Level of evidence:**

Level III. A systematic review of level I – III studies.

## 1 Introduction

Surgical site infections (SSIs) in orthopaedic surgery are a devastating postoperative complication primarily owing to the common use of implants. The management of SSIs in orthopaedic surgery often require major revision surgery as well as prolonged antibiotic treatment.^1^ As such, the application of local vancomycin (LV) powder during wound closure has been of recent interest in orthopedic surgery.^2^ Vancomycin is a tricyclic glycopeptide antibiotic that targets specifically gram-positive bacteria by inhibiting cell wall synthesis and is an effective antimicrobial against methicillin-resistant *Staphylococcus* aureus (MRSA).^3^ This is ideal, as SSIs in orthopedics are predominantly caused by *Staphylococcus* and other gram-positive species, with gram-negatives accounting for only 5–30% of infections.^4^ Local use allows for high concentrations of antibiotic at the surgical site with limited adverse systemic effects.^4^

The literature to date has suggested reduced SSIs with local vancomycin administration in spine surgery,^4^ arthroplasty,^5^ and various sports surgeries,^6^ although debate remains for routine use.^7^ For these reasons, local vancomycin is being increasingly studied and used in multiple orthopedic subspecialties. Traditionally, vancomycin has been administered as a topical powder during wound closure or mixed in saline for graft-soaking in anterior cruciate ligament reconstruction. However, several novel modalities have been recently studied, including interosseus administration during total knee arthroplasty (TKA),^8^ vancomycin suspended in saline for use in wound irrigation,^9^^,^^10^ and vancomycin-soaked allografts for high-tibial osteotomies (HTO).^11^

Disadvantages of the routine administration of vancomycin for the prevention of SSIs include adverse systemic reactions, cost, and the theoretical risk of developing antibiotic resistance. Local vancomycin powder is well-tolerated, as there are only low levels systemically and only a single reported case of anaphylaxis after its use in spinal surgery.^12^ From a cost perspective, the cost of vancomycin powder is low (generally between $2.50 and $44.00)^13^, ^14^, ^15^ whereas the cost of revision surgery can be up to tens of thousands of dollars.^16^ Additionally, there is some systemic absorption of locally applied vancomycin, and these levels are often sub-therapeutic which could theoretically allow bacteria such as *Staphylococcus* to proliferate with a higher potential of developing vancomycin resistance.^17^

Therefore, the use of locally applied intra-articular/intra-wound/soaking of antibiotics as an adjunct to parenteral antibiotics has not become standard for orthopaedics. Although there have been previous systematic reviews addressing LV use within certain subspecialties, there have been no recent systematic reviews evaluating the use of LV within the entire orthopaedic specialty. Therefore, the aim of this review is to assess how local vancomycin impacts the rates and microbiology of surgical site infections.

## 2 Methods

This systematic review and meta-analysis followed the guidelines of the Preferred Reporting Items for Systematic Reviews and Meta-analysis (PRISMA; Fig. I).^18^

**Fig. 1:**
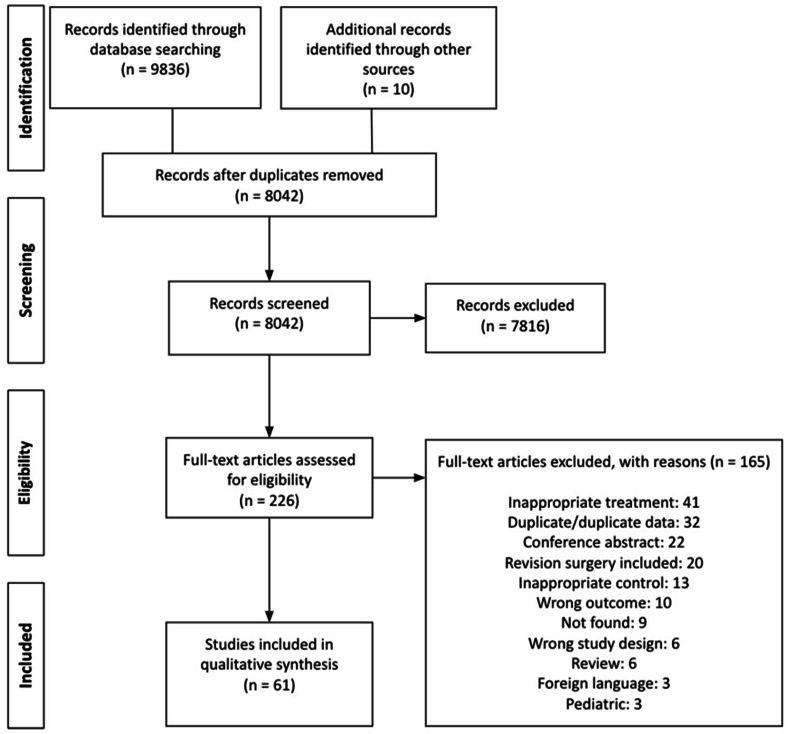
Preferred reporting items for systematic reviews and meta-analysis (PRISMA) diagram.

### 2.1 Comprehensive search strategy

An electronic systematic search of three databases (the Excerpta Medica Database (Embase), the Medical Literature Analysis and Retrieval System Online (Medline), and Web of Science) was performed through August 14, 2022 by two reviewers (DL and JS) for literature related to local vancomycin administration and orthopaedic surgery procedures (APPENDIX I). The inclusion criteria were (1) studies that administered local vancomycin (powder, irrigation, soaking, etc.), (2) comparative studies with at least two arms, (3) adequate control groups and treatment groups that had no other local antimicrobials/antibiotics administered, (4) primary orthopaedic procedures, (5) available in English, (6) adult population, (7) human studies, and (8) all levels of evidence. Exclusion criteria consisted of (1) revision surgical procedures or repeat surgeries in previously operated areas, (2) prior infection, (3) additional antimicrobial adjuncts, (4) no specific infection rates reported, (5) longer-term drug eluting adjuncts (i.e. cement, beads, bone graft, etc.), (6) cadaver/biomechanical studies, and (7) case reports. If two studies reported outcomes on the same patient population, the study with the longest follow-up period was selected and the other study rejected. Similarly, if there was a subsequent study published with a subset of another paper's population, the paper with the subset was eliminated.

### 2.2 Study screening

The titles and abstracts of all identified studies were screened by two authors (DL and JS). All disagreements or incomplete abstracts were advanced to full-text review and reviewed by a third reviewer (HAK). Full-text review followed using the predetermined inclusion and exclusion criteria above by three authors (DL, JS and HAK). Any disagreements were reviewed by a fourth reviewer (AA) who resolved conflicts. A Kappa (κ) score was calculated to determine the level of agreement between each reviewer.^19^

### 2.3 Assessment of study quality

A quality assessment of all nonrandomized comparative studies was completed and averaged by three reviewers (DL, JS, and HAK) using the Methodological Index for Non-Randomized Studies (MINORS) criteria.^20^ The quality assessment of for all randomized control trials was assessed by two authors (DL and JS) using the Cochrane Collaboration's tool for assessing risk of bias.^21^

### 2.4 Data abstraction from included studies

Three reviewers (DL, JS, and HAK) each abstracted data from one-third of the studies while reviewing the accuracy of the other abstracted two-thirds, and vice-versa. Google Sheets (Google, Alphabet Inc.) was used with predetermined tables to abstract and record the data. Study characteristics (authors, journal, study design, publication year, level of evidence (LOE), etc.), patient demographics (number of participants, sex, age, etc.), primary outcome (rate of deep surgical site infection), secondary outcomes (superficial SSIs, general SSI, microbial isolates, etc.), method of deep SSI diagnosis, systemic adverse reactions to vancomycin, and follow-up length was abstracted from the studies if available. We classified the LOE based on the author's statement or based the American Academy of Orthopaedic Surgeons (AAOS) Evidence-Based Practice Committee guidelines if not stated.^22^

### 2.5 Sub-specialties

The included studies were divided based on orthopaedic subspecialty. The Spine sub-specialty included all studies that assessed local vancomycin surgery in primary spine surgery, with or without instrumentation. These studies were limited to operations that are commonly performed by orthopaedic surgery and excluded any spinal cord/intra-dural operations or operations primarily performed by neurosurgeons (insertion of neurostimulators, etc.), and did not include studies focusing primarily on spinal tumor surgery. The Arthroplasty subspecialty included all studies that assessed local vancomycin application for patients undergoing primary total hip arthroplasty (THA), total knee arthroplasty (TKA), and/or unicompartmental knee arthroplasty (UKA). Outcomes were reported in infections per procedure given that some patients had bilateral operations. The Sports Medicine subspecialty included all studies with patients that underwent anterior cruciate ligament reconstruction (ACLR) with autograft or allograft, regardless of graft used. Trauma included all studies that compared the use of local topical administration of vancomycin for open reduction and internal fixation of fractures. ‘Other’ included all studies that did not fit into the above subspecialty sections.

### 2.6 Outcomes

The primary outcome was overall surgical site infections. This included any reported infection; deep, superficial, or other/undefined. The secondary outcomes consisted of deep infections, microbial isolates, and systemic adverse events. Deep infections were defined in differently in the included studies. Definitions of deep surgical site infection were categorized and included: defined as per the Center of Disease Control (CDC) guidelines of deep surgical site infection, as per validated arthroplasty-specific criteria, identified on imaging, infection deep to the fascial layer, an intra-articular infection, positive culture from deep in the wound, infection requiring secondary surgery, an aspiration of an infected collection deep in the surgical site, definition not applicable, defined as per the author's guidelines, or not defined. All instances of deep infection were combined regardless of definition. If not reported, this was not included as a deep infection.

The microbiology of the patients with infections was also abstracted. All reported cultures were recorded. In cases with polymicrobial infections, each bacterium was reported separately based on which isolated microbes were found. If the specific class, name, or identifier of the pathogenic microbe was not reported, this was not included in the analysis. A pie chart was created to reflect the relative proportion of each type of microbe that was isolated from infections. Bacteria were divided into *Staphylococcus aureus* (SA; Methicillin-sensitive, -resistant, or sensitivity not reported), Vancomycin-resistant enterococci (VRE), other gram-positive (OGP; gram-positive bacteria other than SA or VRE, although some papers may not specifically report *S. aureus* or VRE and therefore may include this bacteria in these instances), gram negative bacteria (GNB), culture indeterminate (CI; cultures without growth or unable to determine pathogen), and other (reported as ‘other’ in literature or other pathogens such as *mycobacterium abscessus*).

### 2.7 Statistical analysis

Due to the significant heterogeneity and quality between the included studies with variations in length of follow-up, surgical technique, method of vancomycin application, surgeons, patient populations, we did not pool the data in a meta-analysis.^23^ Instead, all outcomes consistently reported have been presented as ranges of infection rates and are reported in narrative summary fashion. Statistical significance was recorded with the statistical threshold as determined by individual studies.

### 2.8 Human and animal rights

There were no violations of either human or animal rights.

## 3 Results

The initial electronic search from Embase, Medline, and ClinicalTrials.gov yielded 9836 studies and another 10 studies were identified with a manual search. After the exclusion of duplicates, 8042 studies remained, with 226 studies remaining after title and abstract review. Sixty-one studies were included in the final review after full text review^15^^,^^24^, ^25^, ^26^, ^27^, ^28^, ^29^, ^30^, ^31^, ^32^, ^33^, ^34^, ^35^, ^36^, ^37^, ^38^, ^39^, ^40^, ^41^, ^42^, ^43^, ^44^, ^45^, ^46^, ^47^, ^48^, ^49^, ^50^, ^51^, ^52^, ^53^, ^54^, ^55^, ^56^, ^57^, ^58^, ^59^, ^60^, ^61^, ^62^, ^63^, ^64^, ^65^, ^66^, ^67^, ^68^, ^69^, ^70^, ^71^, ^72^, ^73^, ^74^, ^75^, ^76^, ^77^, ^78^, ^79^, ^80^, ^81^, ^82^, ^83^ The full PRISMA diagram can be found in Fig. I. Near-perfect agreement was obtained in both the title and abstract screening (κ = 0.929; 95 % CI, 0.903–0.955) and the full-text screening (κ = 0.884; 0.820–0.948). The baseline study characteristics and MINORS quality assessment score for each study is included in APPENDIX II and the Cochrane risk-of-bias tool for randomized control trials assessment is presented in APPENDIX III. The majority of non-RCT studies were of moderate to low quality. Demographics for all studies can be found in APPENDIX IV.

### 3.1 Spine

There were 26 studies identified that assessed the use of local vancomycin administration (25/26 studies used powder) in spine surgery.^15^^,^^24^, ^25^, ^26^, ^27^, ^28^, ^29^, ^30^, ^31^, ^32^, ^33^, ^34^, ^35^, ^36^, ^37^, ^38^, ^39^, ^40^, ^41^, ^42^, ^43^, ^44^, ^45^, ^46^, ^47^, ^48^ The 26 included studies consisted of 16,148 patients, with 7,946 in the vancomycin treatment group and 8,202 patients without local vancomycin. The majority of studies reported that both groups received a cephalosporin antibiotic pre/intraoperatively (23/26), followed by a cephalosporin for 24–48h postoperatively (18/26; APPENDIX II). The overall infection rates ranged from 0.8% to 15.3% in control groups compared to ranges of 0.0% to8.2% in patients treated with local vancomycin (Table I). For deep infections, the infection rates ranged from 0.5% to 15.1% in controls and 0.0% to 6.4% in treatment groups. Additionally, 15 studies found statistically significant decreased overall infection rates when applying local vancomycin, compared to one study that found significant increases in infections with local application (Table I). Twenty-five studies reported on the microbiology of the surgical infections. In the vancomycin treatment group, the majority of infections were caused by GNB (45.9%) whereas in the control group, SA (42.4%) was most common (FIGURE IIA).

**Table 1:** Spine surgery infections.

Author (Year)	Patients, n	Overall Infections, %	Overall Infections, n	Deep Infections, %	Deep Infections, n	Superficial Infections, n	Other/Undefined Infections, n
Adhikari (2020)							
Control	70	**1.4**	1	**1.4**	1		
Intervention	88	**3.4**	3	**3.4**	3		
Caroom (2013)							
Control	72	**15.3**	11				11
Intervention	40	**0.0**^a^	0				0
Chotai (2017)							
Control	1587	**2.5**	40	**2.5**	40		
Intervention	1215	**1.6**^a^	20	**1.6**^a^	20		
Delgado-López (2020)							
Control	150	**10.7**	16	**6.0**	9	7	
Intervention	150	**8.0**	12	**3.3**	5	7	
do Nascimento (2020)							
Control	47	**8.5**	4				4
Intervention	49	**8.2**	4				4
Haimoto (2018)							
Control	268	**5.6**	15				15
Intervention	247	**0.0**^a^	0				0
Hasan (2020)							
Control	187	**5.6**	11	**5.6**	11		
Intervention	190	**1.4**^a^	2	**1.4**^a^	2		
Hey (2017)							
Control	272	**6.3**	17	**3.7**	10	7	
Intervention	117	**0.9**^a^	1	**0.9**^a^	1	0	
Khanna (2019)							
Control	2521	**0.8**	20				20
Intervention	2354	**1.4**	33				33
Kim (2013)							
Control	34	**14.7**	5	**8.8**	3	2	
Intervention	40	**0.0**^a^	0	**0.0**^a^	0	0	
Kunakornsawat (2019)							
Control	135	**3.0**	4	**3.0**	4		
Intervention	265	**3.4**	9	**3.4**	9		
Lee (2018)							
Control	209	**5.7**	12				12
Intervention	489	**2.0**^a^	10				10
Maajid (2018)							
Control	150	**11.3**	17				17
Intervention	153	**2.6**^a^	4				4
Madhuchandra (2018)							
Control	40	**12.5**	5			0	5
Intervention	40	**2.5**^a^	1			1	0
Mirzashahi (2018)							
Control	187	**2.7**	5	**2.7**	5	0	
Intervention	193	**5.2**	10	**5.2**	10	0	
O'Neill (2011)							
Control	54	**13.0**	7	**9.3**	5	2	
Intervention	56	**0.0**^a^	0	**0.0**	0	0	
Oktay (2021)							
Control	107	**6.5**	7	**3.7**	4	3	
Intervention	102	**2.0**^a^	2	**1.0**	1	1	
Salimi (2022)							
Control	188	**6.9**	13	**3.2**	6	7	
Intervention	187	**6.4**	12	**3.7**	7	5	
Schär (2021)							
Control	17	**5.9**	1	**5.9**	1		
Intervention	17	**0.0**	0	**0.0**	0		
Scheverin (2015)							
Control	281	**5.0**	14	**5.0**	14		
Intervention	232	**1.3**^a^	3	**1.3**^a^	3		
Strom (2013a)							
Control	97	**11.3**	11	**11.3**	11		
Intervention	156	**0.0**^a^	0	**0.0**^a^	0		
Strom (2013b)							
Control	92	**10.9**	10	**10.9**	10		
Intervention	79	**2.5**^a^	2	**2.5**^a^	2		
Takeuchi (2020)							
Control	354	**2.5**	9	**2.5**	9		
Intervention	314	**0.3**^a^	1	**0.3**^a^	1		
Tubaki (2013)							
Control	474	**1.7**	8	**1.3**	6	2	
Intervention	433	**1.6**	7	**1.4**	6	1	
Vakayil (2021)							
Control	221	**2.3**^a^	5	**0.5**^a^	1	4	
Intervention	221	**4.5**	10	**1.4**	3	7	
Wang (2022)							
Control	86	**15.1**	13	**15.1**	13		
Intervention	110	**6.4**^a^	7	**6.4**^a^	7		

a Denotes statistically significant difference (P ≤ 0.05).

**Fig. 2:**
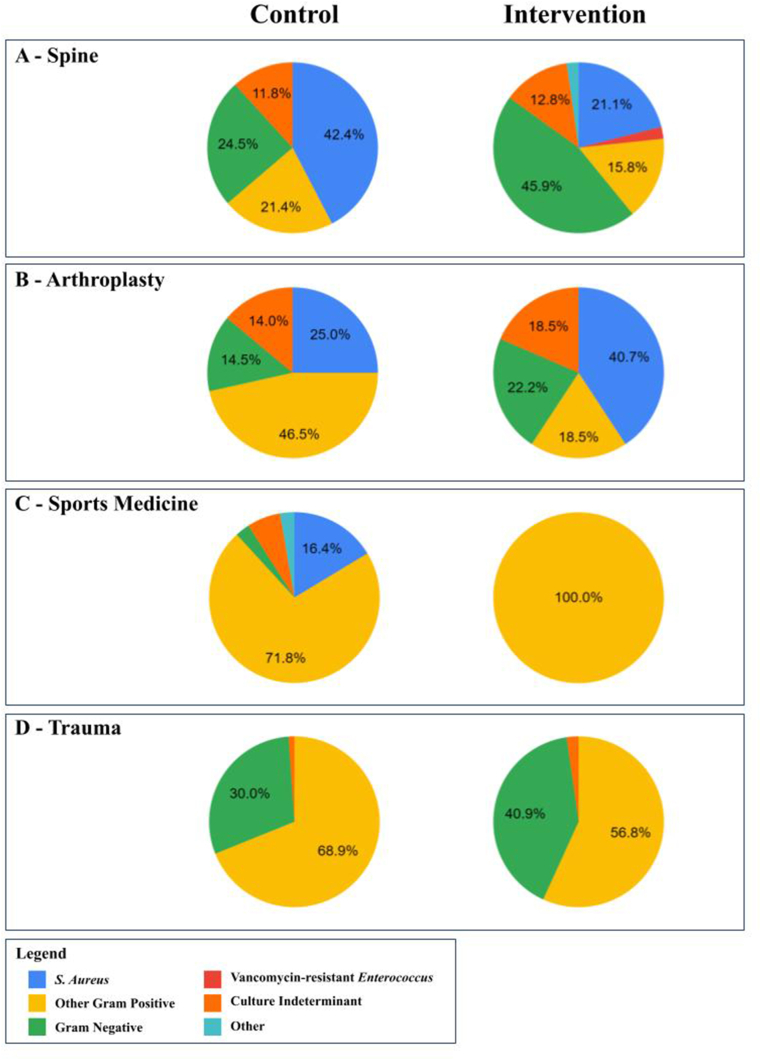
Microbiology of Reported Infections. A – Reported microbial infections in spine surgery. B - Reported microbial infections in arthroplasty surgery. C - Reported microbial infections in sports medicine surgery. D- Reported microbial infections in trauma surgery.

### 3.2 Arthroplasty

Fourteen studies (18,405 procedures) were identified that assessed the use of local vancomycin administration in hip and/or knee arthroplasty, 6,717 procedures including local vancomycin administration and 11,688 without.^40^^,^^49^, ^50^, ^51^, ^52^, ^53^, ^54^, ^55^, ^56^, ^57^, ^58^, ^59^, ^60^^,^^62^ The majority of studies reported that both groups received a cephalosporin antibiotic pre/intraoperatively (10/14), followed by a cephalosporin for 24–48h postoperatively (8/14; APPENDIX II). The overall infection rates ranged from 0.0% to 13.4% in control groups compared to ranges of 0.0% to 9.8% in local vancomycin groups (Table II). For deep infections, the infection rates ranged from 0.0% to 9.4 % in controls and 0.0% to 7.8% in treatment groups. Additionally, 5 studies found statistically significant decreased overall infection rates when applying local vancomycin (Table II). In the vancomycin treatment group, most infections were caused by SA (40.7%), compared to OGP (46.5%) in the control group (FIGURE IIB).

**Table 2:** Arthroplasty infections.

Author (Year)	Procedures, n	Overall Infections, %	Overall Infections, n	Deep Infections, %	Deep Infections, n	Superficial Infections, n	Other/Undefined Infections, n
Aljuhani (2021)							
Control	49	**2.0**	1	**2.0**	1		
Intervention	49	**0.0**	0	**0.0**	0		
Assor (2010)							
Control	73	**6.8**	5	**4.1**	3	2	
Intervention	62	**1.6**	1	**0.0**	0^a^	1	
Cohen (2019)							
Control	246	**1.6**	4				4
Intervention	309	**0.6**	2				2
Crawford (2018)							
Control	815	**1.5**	12	**0.9**	7	5	
Intervention	1070	**0.5**^a^	5	**0.1**^a^	1	4	
Dial (2018)							
Control	128	**8.6**	11	**5.5**	7	2	2
Intervention	127	**1.6**	2	**0.8**^a^	1	1	
Duan (2022)							
Control	1018	**2.5**	25	**1.9**	19	6	
Intervention	1175	**0.1**^a^	1	**0.0**^a^	0	1	
Hanada (2019)							
Control	92	**7.6**	7	**7.6**	7		
Intervention	110	**4.5**	5	**4.5**	5		
Khatri (2017)							
Control	64	**12.5**	8	**9.4**	6	2	
Intervention	51	**9.8**	5	**7.8**	4	1	
Klasan (2021)							
Control	331	**0.0**	0	**0.0**	0		
Intervention	301	**0.3**	1	**0.3**	1		
Koutalos (2020)							
Control	148	**4.1**	6	**0.7**	1	1	4
Intervention	142	**2.1**	3	**1.4**	2	0	1
Matziolis (2020)							
Control	7863	**1.2**	92				92
Intervention	1082	**0.4**^a^	4				4
Tahmasebi (2021)							
Control	314	**13.4**	42	**1.9**	6	36	
Intervention	1710	**2.3**^a^	39	**0.4**^a^	7	32	
Wu (2022)							
Control	45	**8.9**	4	**8.9**	4		
Intervention	45	**0.0**^a^	0	**0.0**^a^	0		
Yavuz (2020)							
Control	502	**1.0**	5	**1.0**	5		
Intervention	474	**0.8**	4	**0.8**	4		

a Denotes statistically significant difference (P ≤ 0.05).

### 3.3 Sports medicine

There were 11 studies (26,985 patients) identified that compared the use of local vancomycin administration in anterior cruciate ligament reconstruction.^63^, ^64^, ^65^, ^66^, ^67^, ^68^, ^69^, ^70^, ^71^, ^72^, ^73^ All studies used autografts and 9,564 patients had autografts soaked in vancomycin compared to 17,331 patients without. All studies reported that both groups received a cephalosporin antibiotic pre/intraoperatively, and the majority did not report postoperative antibiotics (10/11; APPENDIX II). The overall infection rates ranged from 0.3% to 2.4% in control groups compared to 0.0 % in vancomycin soaking groups (Table III). For deep infections, the infection rates ranged from 0.3 % to 2.4 % in controls and was 0.0 % in treatment groups. Additionally, 10 studies found statistically significant decreased overall infection rates when applying local vancomycin (Table III). In the vancomycin treatment group, the bacteria isolated from the single infection was *S.* caprae (OGP group), whereas the majority of infections in the control group were OGP (71.8%; FIGURE IIC).

**Table 3:** Sports medicine infections.

Author (Year)	Procedures, n	Overall Infections, %	Overall Infections, n	Deep Infections, %	Deep Infections, n	Superficial Infections, n	Other/Undefined Infections, n
Banios (2021)							
Control	1242	**0.6**	7	**0.6**	7		
Intervention	593	**0.0**^a^	0	**0.0**^a^	0		
Bohu (2020)							
Control	1184	**0.6**	7	**0.6**	7		
Intervention	490	**0.0**	0	**0.0**	0		
Carrozzo (2022)							
Control	3228	**0.3**	11	**0.3**	11		
Intervention	2072	**0.0**^a^	1	**0.0**^a^	1		
Figueroa (2019)							
Control	230	**1.7**	4	**1.7**	4		
Intervention	260	**0.0**^a^	0	**0.0**^a^	0		
Hees (2022)							
Control	636	**1.6**	10	**1.6**	10		
Intervention	536	**0.0**^a^	0	**0.0**^a^	0		
Offerhaus (2019)							
Control	926	**2.4**	22	**2.4**	22		
Intervention	853	**0.0**^a^	0	**0.0**^a^	0		
Pérez-Prieto (2016)							
Control	810	**1.9**	15	**1.9**	15		
Intervention	734	**0.0**^a^	0	**0.0**^a^	0		
Pérez-Prieto (2021)							
Control	383	**1.3**	5				5
Intervention	402	**0.0**^a^	0				0
Phegan (2015)							
Control	285	**1.4**	4	**1.4**	4		
Intervention	1300	**0.0**^a^	0	**0.0**^a^	0		
Schuster (2020)							
Control	10,516	**0.3**	35	**0.3**	35		
Intervention	2277	**0.0**^a^	0	**0.0**^a^	0		
Wan (2020)							
Control	185	**1.6**	3	**1.6**	3		
Intervention	122	**0.0**^a^	0	**0.0**^a^	0		

a Denotes statistically significant difference (P ≤ 0.05).

### 3.4 Trauma

There were five studies (3,198 patients) identified that assessed the use of local vancomycin administration in trauma open reduction internal fixation (ORIF), with 884 patients receiving vancomycin and 2,314 patients not.^74^, ^75^, ^76^, ^77^, ^78^ Two studies reported that both groups received a cephalosporin antibiotic pre/intraoperatively (2/5), followed by an institution specific postoperative regimen (3/5; APPENDIX II). The overall infection rates ranged from 8.3% to 17.7% in control groups compared to ranges of 0.0% to 12.8 % in patients treated with local vancomycin (Table IV). For deep infections, the infection rates ranged from 6.1% to 11.4% in controls and 0.0% to 10.7% in treatment groups. Additionally, two studies found statistically significant decreased overall infection rates when applying local vancomycin, whereas three studies found significant decreases in deep infections with vancomycin added (Table IV). In the vancomycin treatment group, most infections were caused by OGP (56.8%), compared to a larger majority of OGP infections (68.9%) in the control group (FIGURE IID).

**Table 4:** Trauma infections.

Author (Year)	Procedures, n	Overall Infections, %	Overall Infections, n	Deep Infections, %	Deep Infections, n	Superficial Infections, n	Other/Undefined Infections, n
Balabanova (2021)							
Control	318	**9.7**	31	**9.7**	31		
Intervention	28	**10.7**	3	**10.7**	3		
Cichos (2021)							
Control	326	**8.3**	27	**6.1**	20	7	
Intervention	294	**6.8**	20	**6.1**	18	2	
O'Toole (2021)							
Control	481	**17.7**	85	**10.0**	48	14	23
Intervention	499	**12.8**	64	**6.0**^a^	30	17	17
Qadir (2021)							
Control	783	**11.4**	89	**11.4**	89		
Intervention	35	**0.0**^a^	0	**0.0**^a^	0		
Vaida (2022)							
Control	388	**9.3**	36	**9.3**	36		
Intervention	46	**8.7**^a^	4	**8.7**^a^	4		

a Denotes statistically significant difference (P ≤ 0.05).

### 3.5 Other surgery

There were five other studies identified that assessed the use of local vancomycin administration in orthopaedic surgery procedures during oncologic procedures,^79^ hip hemiarthroplasty for treating hip fractures,^80^ high tibial osteotomies,^81^ spine tumor surgery,^82^ and in foot and ankle surgery.^83^ All studies reported that both groups received a cephalosporin antibiotic pre/intraoperatively, followed by different postoperative regimens (APPENDIX II). In the vancomycin treated patients, there were 16 deep infections and four superficial infections, with 28 overall infections, compared to the control patients which had 29 deep infections and 14 superficial infections, with 54 overall infections (Table V). One study found statistically significant decreased overall and deep infection rates when applying local vancomycin (Table V).

**Table 5:** Other infections.

Author (Year)	Procedures, n	Overall Infections, %	Overall Infections, n	Deep Infections, %	Deep Infections, n	Superficial Infections, n	Other/Undefined Infections, n
Byregowda (2017)							
Control	221	**7.7**	17	**7.7**	17		
Intervention	254	**5.5**	14	**5.5**	14		
Erken (2020)							
Control	58	**6.9**	4				4
Intervention	35	**5.7**	2				2
Koh (2022)							
Control	50	**22.0**	11	**4.0**	2	9	0
Intervention	64	**3.1**	2	**0.0**	0	2	0
Mesfin (2019)							
Control	47	**14.9**	7				7
Intervention	54	**11.1**	6				6
Wukich (2015)							
Control	81	**18.5**	15	**12.3**	10	5	
Intervention	81	**4.9**^a^	4	**2.5**^a^	2	2	

a Denotes statistically significant difference (P ≤ 0.05).

## 4 Discussion

This is the first study to present the available data for studies comparing local vancomycin use to control in all the subspecialties of orthopaedic surgery for primary operations. In the present study, there was a trend for local vancomycin administration to decrease overall and deep infection rates within primary orthopaedic spine, arthroplasty, sports medicine, and trauma surgery. There were 15 studies (of 26) in spine surgery, five (of 14) in arthroplasty, ten (of 11) in sports medicine, and two (of five) in trauma surgery that found statistically significant decreases in overall infection rates when applying local vancomycin. However, given the significant heterogeneity within the included studies, there should be caution when applying these findings to clinical practice. The microbiology for patients treated with vancomycin favoured decreased gram-positive organisms and greater proportions of gram-negative organisms in spine, arthroplasty, and trauma surgery, however absolute numbers of GNB remained comparable. There were no reported systemic adverse events attributed to local vancomycin use.

### 4.1 Antimicrobial stewardship

While this study has demonstrated that locally applied intra-wound vancomycin appears to favor decreased infection rates in many orthopaedic surgeries, whether this represents good practice and antibiotic stewardship has not been discussed in great depth. With over 28 million orthopedic surgeries performed each year,^84^ one must always consider the theoretical risk of developing antibiotic resistant organisms.

According to the Centers for Disease Control and Prevention (CDC), antimicrobial resistance is an urgent global public health issue with nearly 2.8 million antimicrobial resistant (AR) infections a year.^85^^,^^86^ Excessive use of antibiotics can allow organisms to adapt. Antibiotic Stewardship Programs (ASPs) therefore have been created, comprising multidisciplinary teams set up to minimize the excess unnecessary use of antimicrobials^87^ - the choice, recommend doses, duration and costs thereof.

While our study suggests local vancomycin administration may decrease infection in orthopaedic surgery, the concerns for whether the practice remains good stewardship are due to the following factors.^88^

1. The insufficient exposure time between the microorganisms and the antimicrobial agent
2. the potential toxicity or adverse reactions of the antibiotics
3. the potential for the development of antibiotic resistance

In experimental studies, it has been suggested that from the time of administration to maximal effectivity is 3h in terms of bacterial suppression.^89^ However, from a pharmocokinetic point of view, the studies in spine surgery show that after administration of 2g of vancomycin powder, the local levels of vancomycin peak at 1500 mg/L at day 1. Even at the third day, they remain present at 100 mg/L.^90^ So this suggests that there is good antibiotic exposure time.

The danger is if these levels transfer to the blood and therefore have systemic effects. A recent study by O'Toole et al.^91^ found that patients given 1g of topical vancomycin powder during high-risk tibial fracture management had detectable although low (<5ug/mL) serum levels at an hour and at 6–8 h after surgery, whereas the therapeutic level is 12–15ug/mL.^91^ These levels were not significant enough to cause/raise concerns for typical side effects of vancomycin such as nephrotoxicity or ototoxicity. It is therefore unlikely that these local antibiotics are reaching serum levels sufficient to cause adverse effects, and there were no reported systemic events in any of the included studies in this review.

Finally, is the concern of microbial resistance. There is no significant evidence for this, but it has been shown in some studies that topical application of antimicrobial prophylaxis, can lead to “selective pressures on wound flora”.^92^ As such, levels >10ug/mL are recommended by the infectious disease association of America to avoid development of resistance.^93^ Given there is subtherapeutic systemic absorption of locally applied vancomycin, and these drug levels are often unable to inhibit bacterial growth such as *S. aureus*, bacteria could proliferate while being exposed to these low levels of vancomycin and could theoretically develop vancomycin resistance.^91^ While this has been shown in in-vitro models, no real world/clinical data has shown an increase in vancomycin resistance.^94^

There has also been concern that when vancomycin powder was placed into wounds, there would be a decrease in gram positive infections but these would be supplanted by an increase in gram-negative and polymicrobial SSIs.^95^ However, for spine surgery in the present study, although the proportion of GNB infections increased, the absolute number of reported GNB infections remained similar with 61 (out of 7,512 patients in studies that reported infectious microbiology) in the intervention group and 56 (out of 7,741 patients) in the control. Therefore, local vancomycin application in spine surgery likely prevents gram positive bacterial infections without significantly increasing the risk of GNB infections. There was also a small increase in reported VRE in the vancomycin spine surgery treatment group (2.3% of infections), although there were none reported in the control groups. However, caution should be used when interpreting the microbiology results as there was heterogenous reporting of microbial isolates (see limitations).

### 4.2 Current guidelines

While antibiotic stewardship remains an important priority, the societies and governmental associations such as the CDC, the Society for Healthcare Epidemiology for America (SHEA) and the American Hospital Association, there has not been any official guidelines set by any of these organizations.

It is this group's assertion that we as orthopedic surgeons should engage with the associations above for an impartial review.

Until then, while the evidence from this systematic review seems to indicate that orthopedic procedures do benefit from topical antibiotic application. The question remains, “just because we can – does it mean we should?”.

### 4.3 Limitations

There are multiple limitations of this study. The first limitation is the definitions studies used to differentiate deep and superficial infections. Not all papers defined deep infections in the same manner; some grouped deep with superficial infections, and some only reported overall infections. Additionally, some studies only reported deep infections, and when calculating overall SSIs, this could sway the outcome as rates of deep infections based on treatment group could have an outsized impact on overall SSI rates. Secondly, given the method used to compare causative bacteria in infections, absolute numbers of infections should be evaluated with caution as some papers did not report specific bacteria and/or only reported certain common types of bacteria. Lastly, the follow-up periods of some studies were relatively short (i.e. months). This may not encompass all infections, as latent slow growing organisms may take longer to be identified. To better address the theoretical concern of long-term antibiotic resistance, the follow up periods would need to extend much longer and should also be evaluated on a larger scale (i.e. hospital/community pathogenic infections proportions) which was not addressed in this paper.

## 5 Conclusion

Applying local vancomycin during primary orthopaedic surgery procedures may reduce the rates of infections in multiple different orthopaedic specialties, particularly in spine surgery and sports medicine. However, careful consideration should be applied when administering local vancomycin during specific orthopaedic procedures given the heterogeneity of included studies and breadth of surgeries included in this review.

## Ethical statement

There were no ethical concerns associated with the completion of this study as this review solely used previously published data.

## Funding statement

No funding was obtained for the creation of this systematic review.

## Declaration of patient consent form

This systematic review was completed with previously published patient data. No patient consent was required.

## CRediT authorship contribution statement

**Darius L. Lameire:** MD Role, Data curation, Project administration, Writing – original draft. **Jack Soeder:** MD Role, Data curation, Writing – original draft. **Hassaan Abdel Khalik:** MD, MMI Role, Data curation, Writing – original draft. **Ellie Pinsker:** PhD Role, Methodology, Formal analysis. **Nipun Atri:** MD Role, Writing – original draft. **Amir Khoshbin:** MD, MSc, FRCSC Role, Conceptualization, Writing – review & editing. **Lenny Radomski:** MD, FRCSC Role, Conceptualization, Writing – review & editing. **Amit Atrey:** MD, MRCS, FRCSC Role, Conceptualization, Project administration, Writing – review & editing.

## Figures and Tables

**Table:** 

Embase	Medline	Web of Science
1 exp Spine/or spine.mp. 2 exp Spinal Fusion/or spinal.mp. or exp Spinal Fractures/3 orthop*.mp. 4 sport*.mp. or exp Sports/5 exp Knee Injuries/or exp Anterior Cruciate Ligament Injuries/or exp Anterior Cruciate Ligament Reconstruction/or anterior cruciate.mp. or exp Ligaments, Articular/or exp Anterior Cruciate Ligament/or exp Knee Joint/6 arthroscopy.mp. or exp Arthroscopy/7 exp Shoulder Joint/or Arthroscop*.mp. or exp Rotator Cuff Injuries/8 exp Posterior Cruciate Ligament/or posterior cruciate.mp. 9 exp Hip Joint/or exp Osteoarthritis, Hip/or exp "Developmental Dysplasia of the Hip"/or exp Arthroplasty, Replacement, Hip/or exp Hip Dislocation/or exp Hip/or exp Hip Fractures/10 exp Knee/or exp Arthroplasty, Replacement, Knee/or exp Knee Dislocation/11 ankle.mp. or exp Lateral Ligament, Ankle/or exp Ankle Joint/or exp Ankle/or exp Ankle Injuries/or exp Ankle Fractures/or exp Arthroplasty, Replacement, Ankle/12 exp Foot/or foot.mp. or exp Foot Injuries/13 tibia.mp. or exp Tibia/14 exp Arthroplasty, Replacement/or exp Arthroplasty, Replacement, Knee/or exp Arthroplasty, Replacement, Elbow/or arthroplasty.mp. or exp Arthroplasty, Replacement, Hip/or exp Arthroplasty, Replacement, Finger/or exp Arthroplasty, Replacement, Shoulder/or exp Arthroplasty, Replacement, Ankle/or exp Arthroplasty/15 exp Joint Dislocations/or exp Fracture Fixation, Internal/or exp Humeral Fractures/or open reduction.mp. or exp Fractures, Bone/16 exp Femur/or femur.mp. 17 exp Pelvis/or pelvi*.mp. 18 exp Shoulder Injuries/or exp Shoulder/or exp Shoulder Fractures/or exp Shoulder Joint/or shoulder.mp. or exp Shoulder Dislocation/or exp Shoulder Impingement Syndrome/19 elbow.mp. or exp Elbow/20 exp Wrist Injuries/or wrist.mp. or exp Wrist/or exp Wrist Joint/21 exp Radius Fractures/or exp Radius/or radius.mp. 22 1 or 2 or 3 or 4 or 5 or 6 or 7 or 8 or 9 or 10 or 11 or 12 or 13 or 14 or 15 or 16 or 17 or 18 or 19 or 20 or 21 23 surg*.mp. 24 22 and 23 25 exp vancomycin/or vancomyc*.mp. 26 24 and 25	1 exp Spine/or spine.mp. 2 exp Spinal Fusion/or spinal.mp. or exp Spinal Fractures/3 orthop*.mp. 4 sport*.mp. or exp Sports/5 exp Knee Injuries/or exp Anterior Cruciate Ligament Injuries/or exp Anterior Cruciate Ligament Reconstruction/or anterior cruciate.mp. or exp Ligaments, Articular/or exp Anterior Cruciate Ligament/or exp Knee Joint/6 arthroscopy.mp. or exp Arthroscopy/7 exp Shoulder Joint/or Arthroscop*.mp. or exp Rotator Cuff Injuries/8 exp Posterior Cruciate Ligament/or posterior cruciate.mp. 9 exp Hip Joint/or exp Osteoarthritis, Hip/or exp "Developmental Dysplasia of the Hip"/or exp Arthroplasty, Replacement, Hip/or exp Hip Dislocation/or exp Hip/or exp Hip Fractures/10 exp Knee/or exp Arthroplasty, Replacement, Knee/or exp Knee Dislocation/11 ankle.mp. or exp Lateral Ligament, Ankle/or exp Ankle Joint/or exp Ankle/or exp Ankle Injuries/or exp Ankle Fractures/or exp Arthroplasty, Replacement, Ankle/12 exp Foot/or foot.mp. or exp Foot Injuries/13 tibia.mp. or exp Tibia/14 exp Arthroplasty, Replacement/or exp Arthroplasty, Replacement, Knee/or exp Arthroplasty, Replacement, Elbow/or arthroplasty.mp. or exp Arthroplasty, Replacement, Hip/or exp Arthroplasty, Replacement, Finger/or exp Arthroplasty, Replacement, Shoulder/or exp Arthroplasty, Replacement, Ankle/or exp Arthroplasty/15 exp Joint Dislocations/or exp Fracture Fixation, Internal/or exp Humeral Fractures/or open reduction.mp. or exp Fractures, Bone/16 exp Femur/or femur.mp. 17 exp Pelvis/or pelvi*.mp. 18 exp Shoulder Injuries/or exp Shoulder/or exp Shoulder Fractures/or exp Shoulder Joint/or shoulder.mp. or exp Shoulder Dislocation/or exp Shoulder Impingement Syndrome/19 elbow.mp. or exp Elbow/20 exp Wrist Injuries/or wrist.mp. or exp Wrist/or exp Wrist Joint/21 exp Radius Fractures/or exp Radius/or radius.mp. 22 1 or 2 or 3 or 4 or 5 or 6 or 7 or 8 or 9 or 10 or 11 or 12 or 13 or 14 or 15 or 16 or 17 or 18 or 19 or 20 or 21 23 vancomyc*.mp. or exp Vancomycin/24 22 and 23	((ALL =(orthopaedic surgery) OR ALL =(spine) OR ALL =(arthroplasty) OR ALL =(arthroscopy) OR ALL =(foot) OR ALL =(ankle) OR ALL =(knee) OR ALL =(hip) OR ALL =(femur) OR ALL =(pelvis) OR ALL =(shoulder) OR ALL =(humerus) OR ALL =(elbow) OR ALL =(radius) OR ALL =(wrist) OR ALL =(hand)) AND ALL=(surgery)) AND ALL=(vancomycin)

**Table:** 

Author (Year)	Country	Study Design	LOE	Preoperative Antibiotics	Postoperative Antibiotics	Intervention	Deep Infection Definition	MINORS
**Spine**								
Adhikari (2020)	Turkey	Retrospective Cohort	III	Cefazolin	Cefazolin x 24 h	Powder	Not defined	15
Caroom (2013)	United States	Prospective Cohort	II	Not specified	Continued x 24 h	Powder	Not defined	18
Chotai (2017)	United States	Retrospective Cohort	III	Cefazolin or clinda*	Cefazolin x 24 h	Powder	Not defined	17.5
Delgado-López (2020)	Spain	Ambispective	III	Cefazolin or vanco*	Cefazolin + Vanco x 24 h	Powder	Deep to fascia	16
do Nascimento (2020)	Brazil	RCT	I	Not specified	None reported	Irrigation	Not defined	N/A
Haimoto (2018)	Canada	Retrospective Cohort	III	Cefazolin or clinda*	None reported	Powder	Not defined	16
Hasan (2020)	Iraq	RCT	I	Cefazolin	Cefazolin x 24 h	Powder	Imaging	N/A
Hey (2017)	Singapore	Retrospective Cohort	III	Cefazolin or vanco*	Cefazolin x 48 h	Powder	CDC guidelines	17
Khanna (2019)	United States	Retrospective Cohort	III	Cephalosporin	Cephalosporin x 24 h	Powder	Deep to fascia	14.5
Kim (2013)	Korea	Retrospective Cohort	III	Cefazolin	Cefazolin x 24 h	Powder	Positive culture	14
Kunakornsawat (2019)	Thailand	Prospective Cohort	II	Cefazolin or clinda*	Dicloxacillin or clinda x 7d	Powder	CDC guidelines	16
Lee (2018)	United States	Retrospective Cohort	III	Cefazolin or tobra*	Cefazolin x 24 h	Powder	Deep to fascia	14.5
Maajid (2018)	India	Retrospective Cohort	III	Cefazolin or vanco*	Cefazolin x 72 h	Powder	Not defined	14.5
Madhuchandra (2018)	India	Prospective Cohort	II	Ceftriaxone and				
sulbactum	Continued x 7-8d	Powder	Not defined	14				
Mirzashahi (2018)	Iran	RCT	I	Cefazolin or clind*	None reported	Powder	CDC guidelines	N/A
O'Neill (2011)	United States	Retrospective Cohort	III	Cefazolin or clinda*	Cefazolin x 24 h	Powder	Imaging	18
Oktay (2021)	Turkey	Retrospective Cohort	III	Cefazolin	Cefazolin x 24 h	Powder	Deep to fascia	16
Salimi (2022)	Iran	RCT	I	Cephalosporin	Cephalosporin x 24 h	Powder	Not defined	N/A
Schär (2021)	Switzerland	RCT	I	Cefuroxime or clinda*	None reported	Powder	Not defined	N/A
Scheverin (2015)	Argentina	Retrospective Cohort	III	Cephalothin	Cephalosporin x 48 h	Powder	Positive culture	16
Strom (2013a)	United States	Retrospective Cohort	III	Cefazolin or vanco*	None reported	Powder	Required revision	15
Strom (2013b)	United States	Retrospective Cohort	III	Cefazolin or vanco*	None reported	Powder	Required revision	15
Takeuchi (2020)	Japan	Retrospective Cohort	III	Not specified	None reported	Powder	CDC guidelines	16
Tubaki (2013)	India	RCT	I	Cephalosporin	Cephalosporin x 48 h	Powder	Not defined	N/A
Vakayil (2021)	United States	Retrospective Cohort	III	Cefalozin or clinda*	Cefazolin x 24 h	Powder	CDC guidelines	17.5
Wang (2022)	China	Prospective Cohort	II	Cephalosporin or clinda*	Continued x 24–48 h	Powder	CDC guidelines	17
**Arthroplasty**								
Aljuhani (2021)	Saudi Arabia	Retrospective Cohort	III	Not reported	Continued 24–48 h	Powder	Not defined	13
Assor (2010)	France	Prospective Cohort	III	Cefazolin	Cefazolin x 48 h	Powder	Intra-articular	18.5
Cohen (2019)	United States	Retrospective Cohort	III	Cefalozin or clinda*	Cefazolin x 24 h	Powder	Not defined	14
Crawford (2018)	United States	Retrospective Cohort	IV	Not specified	None reported	Powder	Intra-articular	11.5
Dial (2018)	United States	Retrospective Cohort	III	Cefazolin or vanco/clinda	Cefazolin x 24 h	Powder	VASC	17
Duan (2022)	China	Retrospective Cohort	III	Cefazolin	Cefazolin x 24 h	Irrigation	Deep to fascia	15.5
Hanada (2019)	Japan	Prospective Cohort	II	Cefazolin	Cefazolin x 24 h	Powder	Intra-articular	20
Khatri (2017)	India	Retrospective Cohort	III	Cefuroxime	Cephalosporin until drain removal	Powder	Not defined	16.5
Klasan (2021)	New Zealand	Retrospective Cohort	III	Cefalozin or clinda*	Cefazolin x 24 h	Interosseous	VASC	17.5
Koutalos (2020)	Greece	Prospective Cohort	II	Not specified	None reported	Powder	VASC	21
Matziolis (2020)	Germany	Retrospective Cohort	III	Cefalozin or clinda*	None reported	Powder	VASC	14
Tahmasebi (2021)	Iran	Retrospective Cohort	III	Not specified	None reported	Powder	VASC	14
Wu (2022)	China	RCT	I	Cefalozin or clinda*	Cefazolin x 24 h	Powder	Author defined	N/A
Yavuz (2020)	Turkey	Retrospective Cohort	III	Cefazolin	None reported	Powder	VASC	17.5
**Sports Medicine**								
Banios (2021)	Greece	Retrospective Cohort	III	Amikacin and				
ceforanide	Cephalosporin	Soaking	Intra-articular	16.5				
Bohu (2020)	France	Retrospective Cohort	III	Cephalothin or vanco*	None reported	Soaking	Intra-articular	18.5
Carrozzo (2022)	France	Case-Control	III	Cefazolin	None reported	Soaking	Intra-articular	19.5
Figueroa (2019)	Chile	Retrospective Cohort	III	Cefazolin or vanco*	None reported	Soaking	Intra-articular	13.5
Hees (2022)	Germany	Retrospective Cohort	III	Cefazolin	None reported	Soaking	Intra-articular	12.5
Offerhaus (2019)	Germany	Retrospective Cohort	III	Cefazolin	None reported	Soaking	Intra-articular	18
Pérez-Prieto (2016)	Spain	Retrospective Cohort	III	Cefazolin or vanco*	None reported	Soaking	Intra-articular	15
Pérez-Prieto (2021)	Spain	Retrospective Cohort	III	Cefazolin or vanco*	None reported	Soaking	Not defined	15
Phegan (2015)	United States	Retrospective Cohort	III	Cephalothin or unspecified*	None reported	Soaking	Intra-articular	14
Schuster (2020)	Germany	Retrospective Cohort	III	Cefazolin or vanco*	None reported	Soaking	Intra-articular	15.5
Wan (2020)	Hong Kong	Retrospective Cohort	III	Cefazolin or vanco*	None reported	Soaking	Intra-articular	15.5
**Trauma**								
Balabanova (2021)	United States	Case-Control	III	Cefazolin or clinda*	Continued x 24 h	Powder	Not defined	18
Cichos (2021)	United States	Retrospective Cohort	III	Not specified	Institution specific	Powder	Deep to fascia	20.5
O'Toole (2021)	United States	RCT	I	Not specified	Institution specific	Powder	CDC guidelines	N/A
Qadir (2021)	United States	Retrospective Cohort	III	Cefazolin or clinda*	Cefazolin x 24 h	Powder	CDC guidelines	18
Vaida (2022)	United States	Retrospective Cohort	III	Complex regimen	Institution specific	Powder	Required revision	13.5
**Other**								
Byregowda (2017)	India	Retrospective Cohort	III	Cefuroxime or vanco*	Cephalosporin until drain removal	Powder	CDC guidelines	16.5
Erken (2020)	Turkey	Retrospective Cohort	III	Cefazolin	None reported	Powder	Not defined	13.5
Koh (2022)	Singapore	Retrospective Cohort	III	Cefazolin or vanco*	None reported	Soaking	Deep to fascia	18
Mesfin (2019)	United States	Case-Control	III	Cephalosporin or vanco/clinda*	Continued x 24 h	Powder	Not defined	14
Wukich (2015)	United States	Retrospective Cohort	III	Cefazolin or vanco*	Cefazolin + Vanco x 24 h	Powder	Other	13.5

Legend: Clinda, clindamycin; CS, cephalosporin; CDC, Center for Disease Control; d, days; h, hours; LOE, level of evidence; RCT, randomized control trial; Vanco-, vancomycin; VASC, vancomycin arthroplasty-specific criteria. For preoperative antibiotics, * denotes alternative antibiotics given if patient allergic to primary option.

**Figure:**
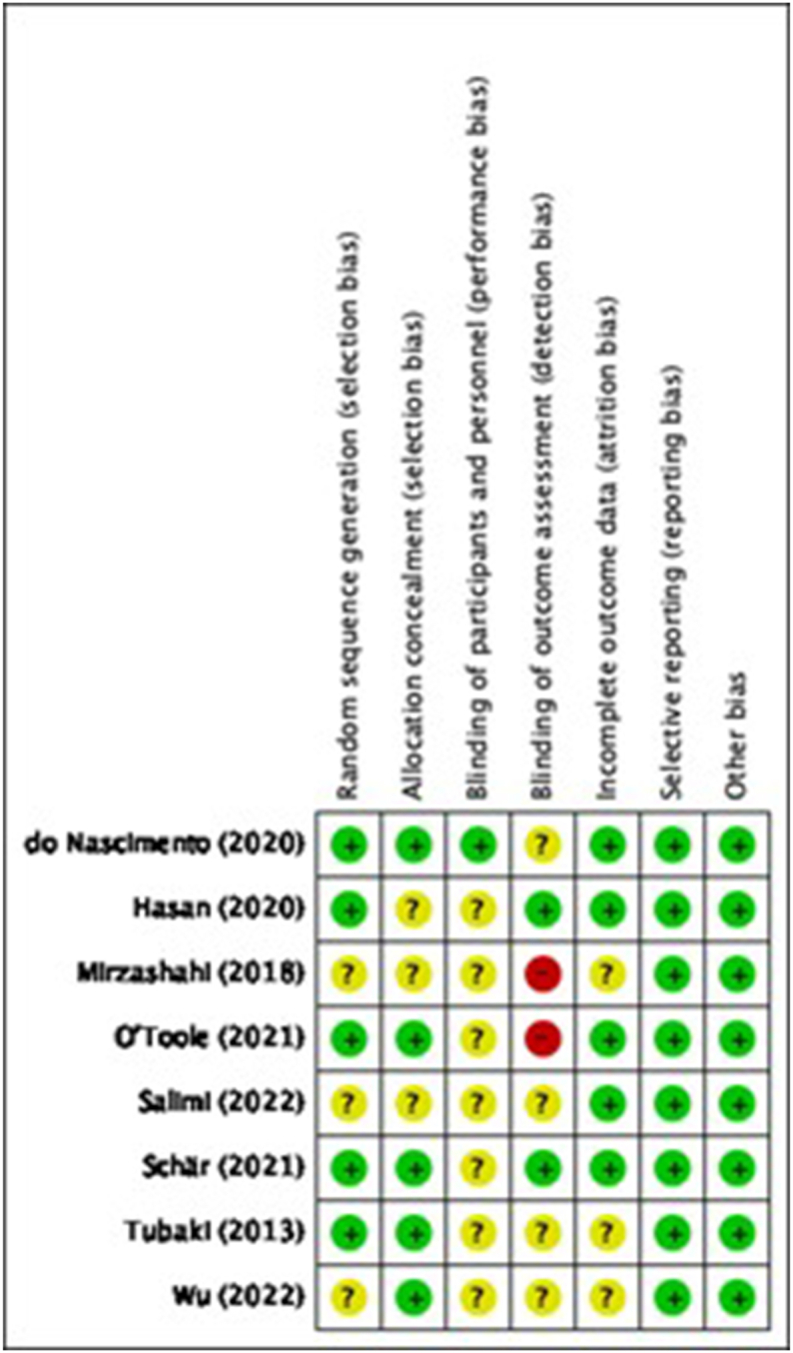


**Table:** 

Author (Year)	Control				Intervention			
	Patients, n	Age, y	Average Follow-Up, Mo (Min Follow-up, Mo)	Female, %	Patients, n	Age, y	Average Follow-Up, Mo (Min Follow-up, Mo)	Female, %
**Spine**								
Adhikari (2020)	70	49.3	NR (12)	62.9 %	88	50.8	NR (12)	54.5 %
Caroom (2013)	72	56.4	NR (6)	NR	40	59.8	NR (6)	NR
Chotai (2017)	1587	NR	NR (12)	NR	1215	NR	NR (12)	NR
Delgado-López (2020)	150	62.0*	4.3 (NR)	41.3 %	150	63.5*	3 (NR)	38.0 %
do Nascimento (2020)	47	45.0*	NR (5)	34.0 %	49	43.0*	NR (5)	18.3 %
Haimoto (2018)	268	54.5	NR (6)	37.3 %	247	58.4	NR (6)	39.2 %
Hasan (2020)	198	34.7	NR (3)	30.8 %	192	38.2	NR (3)	8.8 %
Hey (2017)	272	48.0	NR (12)	53.7 %	117	45.0	NR (12)	43.6 %
Khanna (2019)	2521	58.1	NR (NR)	49.1 %	2354	61.2	NR (NR)	53.1 %
Kim (2013)	34	60.1	NR (NR)	57.5 %	40	57.9	NR (NR)	38.2 %
Kunakornsawat (2019)	135	55.2	NR (NR)	63.7 %	265	52.1	NR (NR)	57.4 %
Lee (2018)	209	56.0	NR (NR)	63.6 %	489	42.0	NR (NR)	56.0 %
Maajid (2018)	150	46.7	NR (3)	46.7 %	153	48.2	NR (3)	41.8 %
Madhuchandra (2018)	40	52.0	NR (NR)	52.5 %	40	50.0	NR (NR)	50.0 %
Mirzashahi (2018)	187	NR	NR (3)	NR	193	NR	NR (3)	NR
O'Neill (2011)	54	45.0	6.9 (0.5)	35.2 %	56	43.0	5.5 (0.5)	37.5 %
Oktay (2021)	107	54.0	NR (3)	57.0 %	102	53.0	NR (3)	61.8 %
Salimi (2022)	188	52.4	NR (3)	56.9 %	187	51.7	NR (3)	56.1 %
Schär (2021)	17	60.5	NR (NR)	41.1 %	17	63.5	NR (NR)	64.7 %
Scheverin (2015)	281	55.0	10 (NR)	48.4 %	232	53.0	10 (NR)	55.1 %
Strom (2013a)	97	64.0	NR (12)	46.0 %	156	64.0	NR (12)	43.0 %
Strom (2013b)	92	60.0	NR (12)	40.2 %	79	60.0	NR (12)	43.0 %
Takeuchi (2020)	354	68.1	NR (NR)	57.1 %	314	69.2	NR (NR)	56.7 %
Tubaki (2013)	474	NR	12.5 (NR)	NR	433	NR	12.2 (NR)	NR
Vakayil (2021)	221	59.3	NR (3)	51.1 %	221	58.8	NR (3)	53.8 %
Wang (2022)	86	53.0	NR (12)	57.1 %	110	56.1	NR (12)	55.6 %
**Arthroplasty**								
Aljuhani (2021)	49^†^	NR	NR (3)	93.9 %	49^†^	NR	NR (3)	73.5 %
Assor (2010)	73^†^	72.0	60 (36)	NR	62^†^	73.0	60 (36)	NR
Cohen (2019)	246^†^	67.3	NR (NR)	55.7 %	309^†^	66.0	NR (NR)	51.8 %
Crawford (2018)	815^†^	63.3	NR (NR)	47.6 %	1,070^†^	64.8	NR (NR)	47.0 %
Dial (2018)	128^†^	61.5	14.4 (3)	50.0 %	137^†^	61.2	8.2 (3)	52.6 %
Duan (2022)	1,018^†^	59.6	NR (3)	53.0 %	1,175^†^	60.0	NR (3)	51.9 %
Hanada (2019)	92^†^	73.3	NR (3)	63.0 %	110^†^	74.6	NR (3)	61.8 %
Khatri (2017)	64^†^	NR	NR (6)	31.3 %	51^†^	NR	NR (6)	37.3 %
Klasan (2021)	331^†^	68.7	NR (12)	57.1 %	301^†^	67.7	NR (12)	58.5 %
Koutalos (2020)	148^†^	68.1	NR (24)	75.0 %	142^†^	66.9	NR (24)	71.1 %
Matziolis (2020)	7,863^†^	68.0	NR (12)	66.6 %	1,082^†^	69.0	NR (12)	62.5 %
Tahmasebi (2021)	314^†^	66.4	NR (12)	80.3 %	1,710^†^	65.0	NR (12)	81.5 %
Wu (2022)	45^†^	68.0	NR (3)	48.9 %	45^†^	67.9	NR (3)	46.7 %
Yavuz (2020)	502^†^	63.4	53.2 (24)	69.3 %	474^†^	65.5	53.2 (24)	68.8 %
**Sports Medicine**								
Banios (2021)	1242	29.1	NR (6)	21.0 %	593	29.9	NR (6)	27.2 %
Bohu (2020)	1184	29.9	NR (12)	32.9 %	490	30.3	NR (12)	35.1 %
Carrozzo (2022)	3228	29.3	NR (12)	28.0 %	2072	28.0	NR (12)	30.0 %
Figueroa (2019)	230	NR	NR (5)	NR	260	NR	NR (5)	NR
Hees (2022)	636	32.9	NR (12)	NR	536	31.4	NR (12)	NR
Offerhaus (2019)	926	32.4	NR (1.5)	38.6 %	853	31.2	NR (1.5)	38.1 %
Pérez-Prieto (2016)	810	NR	NR (NR)	NR	734	NR	NR (NR)	NR
Pérez-Prieto (2021)	383	NR	94 (82)	NR	402	NR	72 (60)	NR
Phegan (2015)	285	30.0	NR (12)	31.6 %	1300	29.0	NR (12)	31.0 %
Schuster (2020)	10,516	NR	NR (1.5)	NR	2277	NR	NR (1.5)	NR
Wan (2020)	185	27.9	NR (9)	16.8 %	120	26.6	NR (9)	20.0 %
**Trauma**								
Balabanova (2021)	318	42.0	NR (6)	40.6 %	28	NR	NR (6)	NR
Cichos (2021)	326	41.5	18 (3)	32.3 %	294	39.6	18 (3)	31.0 %
O'Toole (2021)	481	46.1	NR (6)	36.3 %	499	45.4	NR (6)	37.8 %
Qadir (2021)	783	45.1	NR (6)	32.7 %	35	42.3	NR (6)	37.1 %
Vaida (2022)	388	44.5	NR (2)	33.5 %	46	45.2	NR (2)	45.7 %
**Other**								
Byregowda (2017)	221	NR	NR (12)	NR	254	NR	NR (12)	NR
Erken (2020)	58	81.9	NR (NR)	72.1 %	35	81.9	NR (NR)	57.1 %
Koh (2022)	50	53.0	NR (12)	48.0 %	64	56.0	NR (12)	53.1 %
Mesfin (2019)	47	52.9	NR (1)	33.3 %	54	60.6	NR (1)	51.1 %
Wukich (2015)	81	59.5	8.6 (NR)	40.7 %	81	60.6	6.3 (NR)	40.7 %

Legend: Mo, months; NR, not reported; SD, standard deviation; y, years. *denotes median, ^†^ denotes number of procedures.
